# Adapting reintroduction tactics in successive trials increases the likelihood of establishment for an endangered carnivore in a fenced sanctuary

**DOI:** 10.1371/journal.pone.0234455

**Published:** 2020-06-29

**Authors:** Belinda A. Wilson, Maldwyn J. Evans, William G. Batson, Sam C. Banks, Iain J. Gordon, Donald B. Fletcher, Claire Wimpenny, Jenny Newport, Emily Belton, Annette Rypalski, Tim Portas, Adrian D. Manning

**Affiliations:** 1 Fenner School of Environment and Society, The Australian National University, Canberra, ACT, Australia; 2 Department of Ecosystem Studies, Graduate School of Agricultural and Life Sciences, The University of Tokyo, Tokyo, Japan; 3 National Parks and Wildlife Service, Blue Mountains Branch, Blackheath, NSW, Australia; 4 Charles Darwin University, Darwin, NT, Australia; 5 Central Queensland University, Townsville, QLD, Australia; 6 James Hutton Institute, Craigiebuckler, Aberdeen, Scotland, United Kingdom; 7 CSIRO Land and Water, Townsville, QLD, Australia; 8 ACT Parks and Conservation Service, Canberra, ACT, Australia; 9 The Woodlands and Wetlands Trust, Forde Community Centre, Forde, ACT, Australia; 10 Mt Rothwell Biodiversity Interpretation Centre, Little River, VIC, Australia; 11 Zoo and Wildlife Veterinary Consultancy, Maleny, QLD, Australia; University of Tasmania, AUSTRALIA

## Abstract

Threatened species recovery programs are increasingly turning to reintroductions to reverse biodiversity loss. Here we present a real-world example where tactics (techniques which influence post-release performance and persistence) and an adaptive management framework (which incorporates feedback between monitoring and future actions) improved reintroduction success. Across three successive trials we investigated the influence of tactics on the effective survival and post-release dispersal of endangered eastern quolls (*Dasyurus viverrinus*) reintroduced into Mulligans Flat Woodland Sanctuary, Australian Capital Territory. Founders were monitored for 42 days post-release, and probability of survival and post-release dispersal were tested against trial, origin, sex, den sharing and presence of pouch young. We adopted an adaptive management framework, using monitoring to facilitate rapid learning and to implement interventions that improved reintroduction success. Founders released in the first trial were less likely to survive (28.6%, *n* = 14) than those founders released the second (76.9%, *n* = 13) and third trials (87.5%, *n* = 8). We adapted several tactics in the second and third trials, including the selection of female-only founders to avoid elevated male mortality, and post-mating releases to reduce stress. Founders that moved dens between consecutive nights were less likely to survive, suggesting that minimising post-release dispersal can increase the probability of survival. The probability of moving dens was lower in the second and third trials, for females, and when den sharing with another founder. This study demonstrates that, through iterative trials of tactics involving monitoring and learning, adaptive management can be used to significantly improve the success of reintroduction programs.

## Introduction

**Fig 1:**
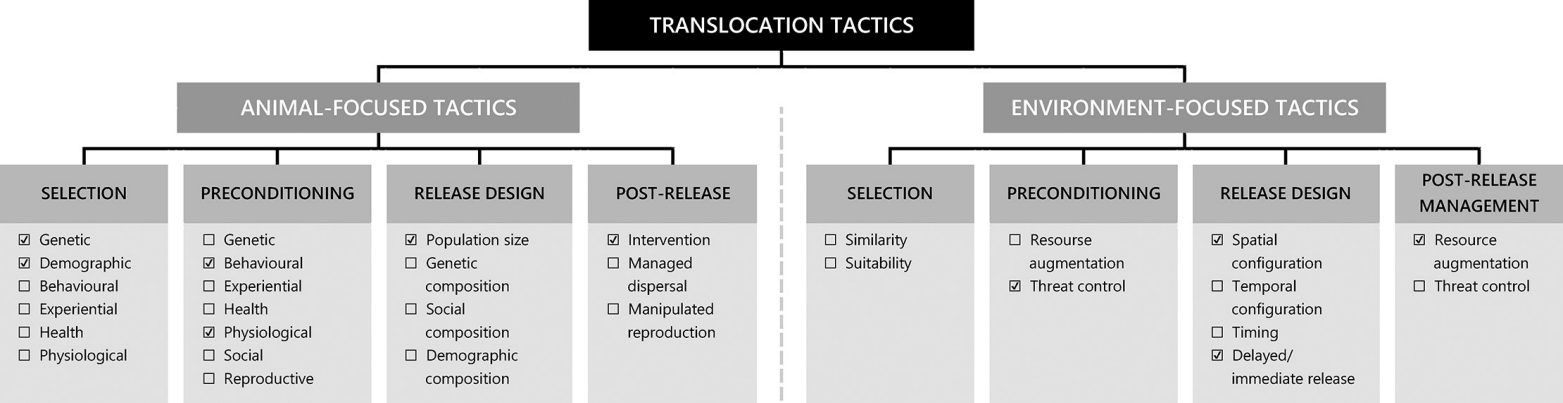
Translocation Tactics Classification System, as adapted from Batson *et al*. (2015). Checked boxes indicate tactics which were employed across the three trial reintroductions of the eastern quoll (*Dasyurus viverrinus*) to Mulligans Flat Woodland Sanctuary, ACT Australia from 2016–18.

Reintroduction describes the intentional movement and release of organisms into their historical range following their local extinction or extirpation, with the aim to re-establish viable, free-ranging populations [1]. Despite their rising popularity as a conservation tool, reintroductions can suffer limited success [2–9]. The success of reintroduction programs can be improved by employing ‘tactics’, defined as techniques which can influence post-release performance and persistence [10]. Tactics may include the selection of founders, release environment and methods [11,12], and the provision of supplementary food and shelter [8]. These tactics should be guided by well-defined objectives termed ‘strategies’, which might include, for example, maximising survival or minimising post-release dispersal [10]. To clarify this thinking, a Translocation Tactics Classification System (TTCS, Fig 1 [10]) was developed to provide a framework to improve the ability to identify, select and design tactics which help achieve defined strategies. The TTCS divides the diversity of tactics by their focus on either the ‘animal’ or the ‘environment’, thereby guiding practitioners through a logical and ecologically relevant framework. By encouraging a standardised and systematic process for designing reintroductions, practitioners can use this tool to rapidly learn from less effective tactics and improve reintroduction success.

Determining which tactics to use can be complex. Reintroductions are often context-specific, and knowledge of the recipient ecosystem is never complete [13]. Adaptive management can address this ‘wicked problem’ [14] by offering a systematic approach to improve management actions through learning from outcomes (‘learning by doing’, [13,15]). Rather than waiting until enough is known about an ecosystem [16], practitioners can implement and adjust management actions ‘on the fly’ in response to outcomes observed through well-designed monitoring. In reintroductions, this can translate to conducting trials, characterised by low replication and control [17], rather than rigid experiments with large sample sizes. This is especially pertinent when dealing with threatened species where inherently small numbers of founding individuals are available. This pragmatic approach can uncover unexpected and valuable results (e.g. [18,19] and [20] case studies), which can inform future trials or feed into full experimental reintroductions. In the face of uncertainty, trial reintroductions can be of greater value than experiments when applied within an adaptive management framework [17].

Here we investigate the effect of tactics employed within an adaptive management framework using a series of three trials for the reintroduction of a locally extinct, marsupial carnivore (the eastern quoll, *Dasyurus viverrinus*) to mainland Australia. Reintroduction success was compared for three cohorts of eastern quolls that were reintroduced to a predator-proof sanctuary in the Australian Capital Territory (ACT) over three years. While survival has a clear influence on reintroduction success, because high mortality can greatly compromise establishment and genetic diversity [21], post-release dispersal is also a crucial consideration [21–23]. Founders that disperse long distances from the release site often have higher mortality rates and are less likely to contribute to effective population size [24], and this dispersal can be impacted by pre-release experience, release method, sex, origin and sociality [5,25–30]. Understanding the tactics that influence this process is key to reducing mortality rates, especially in the establishment phase of a reintroduction. Therefore, we employed tactics within the strategy of maximising survival and minimising post-release dispersal, guided by the TTCS (Fig 1). We asked two questions:

1. Did adapted tactics improve reintroduction outcomes?
2. What mechanisms drove improvements to reintroduction outcomes?

## Materials and methods

### Study area

Mulligans Flat Woodland Sanctuary (MFWS) is a 485 ha reserve containing critically endangered yellow-box *Eucalyptus melliodora* and Blakely’s red gum *Eucalyptus blakelyi* grassy woodland [31] and is situated in north-east Canberra, ACT Australia (-35.166543, 149.157946). MFWS is enclosed by predator-proof fencing to exclude non-native animals such as red foxes (*Vulpes vulpes*), cats (*Felis catus*), European rabbits (*Oryctolagus cuniculus*) and European hares (*Lepus europaeus*), which have been eradicated within the exclosure. The MFWS fence design includes a ‘floppy top’ which prevents introduced predators from climbing into the sanctuary but does not prevent animals from climbing out into the surrounding landscape. MFWS, and the adjoining Goorooyarroo Nature Reserve, are used as an ‘outdoor laboratory’ and form the location of the Mulligans Flat-Goorooyarroo Woodland Experiment (MFGO Experiment, www.mfgowoodlandexperiment.org.au). The experiment aims to restore biodiversity and ecological function to this critically endangered box-gum grassy woodland community [32,33].

### Study species

The eastern quoll (‘murugun’ in Ngunnawal language, [34]) is a small- to medium-sized marsupial [35] which previously inhabited the south-eastern states of Australia. It was last seen on the mainland in 1967 [36]; its extinction has been attributed to predation and competition by introduced predators, habitat loss, disease and human encroachment [37–39]. It is listed as ‘endangered’ by the IUCN [40] and the Commonwealth of Australia (Environment Protection and Biodiversity Conservation Act 1999), and is restricted to Tasmania where it is common in the drier, eastern half of the island State [41,42]. Eastern quolls are often associated with forest-pasture ecotones that provide open grasslands for foraging during the night, and forest habitat where they can den in hollow logs, rocky outcrops and underground burrows during the day [43]. They are nocturnal predators and scavengers, with a diet dominated by invertebrates, as well as occasional birds, small mammals, reptiles, fruit, and carrion [43–45]. The species is sexually dimorphic with a mean adult body mass of 1250 g (min 900—max 2000 g) for males and 805 g (min 700—max 1100 g) for females [38,43]. Males have larger home ranges (mean 44 ha) than do females (mean 35 ha, [37,43]). Females are seasonally polyoestrous and can carry a single litter of up to six young per year [43]. Annual mortality is high, with 20–58% of juveniles surviving to their first breeding season, and life expectancy is 3–4 years [43].

### Founders

We reintroduced the eastern quoll into MFWS in a series of three trials over three years (Trial 1 in 2016, Trial 2 in 2017, and Trial 3 in 2018). To maximise genetic diversity, founders in the Trials 1 and 2 were selected from both captive-bred and wild populations, and in Trial 3 only wild founders were selected. Captive founders were sourced from Mount Rothwell Biodiversity Interpretation Centre (Mt Rothwell), situated 60 km south-west of Melbourne. Wild founders were derived from free-ranging populations across 14 geographic regions in Tasmania, separated by at least 15 km or a significant geographical barrier to eastern quoll dispersal. To minimise impacts on the source population and maximise genetic diversity in the reintroduced population, no more than two animals in each cohort originated from any one site.

### Pre-release health assessments

We selected founders that were in fair to excellent body condition (using a subjective assessment of fat and muscle stored between the hips and spine, see [46]), weighed more than 640g, and were estimated to be 1–2 years old (inferring from tooth condition and wear). They were translocated to the ACT by air and road, where they were anaesthetised and assessed for health and disease (as described in [47]). Founders were microchipped (each animal was identified using a four-character microchip code, see S1 Table) and fitted with VHF collars (32g, V6C 163 Zilco, Sirtrack Ltd, Hawkes Bay, New Zealand) or GPS collars (38g, LiteTrack 30 RF, Sirtrack Ltd, Hawkes Bay, New Zealand). Scat, fur, blood and ear (for DNA extraction) samples were collected.

Translocations were carried out under licenses from the Tasmanian Department of Primary Industries, Parks, Water and Environment (DPIPWE, permits TFA 16025 and 17091, export licences 12818/16 and 13528/17), Victorian Department of Environment, Land, Water and Planning (permit 14505167), and ACT Territory and Municipal Services (import licence L120161261). Reintroduction procedures were approved by The Australian National University Animal Experimentation Ethics Committee (protocol A2016/02).

### Post-release monitoring

Founders were monitored using VHF collars in Trials 1 and 2 and VHF-enabled GPS collars in Trial 3. Survival and den location were monitored daily for 42 days post-release (the ‘establishment period’) because survival plateaued after this period in Trial 1. We removed collars from males after this period and from females after their young had dispersed. We located collars immediately if a mortality signal was detected and conducted necropsies on all deceased animals that could be located.

We conducted post-release health checks every two weeks, though timing and frequency varied due to the reproductive stage of females, weight fluctuations (influencing collar fit), logistical constraints, and ability to re-trap the targeted animal. We conducted all trapping with wire cage traps (31 cm x 31 cm x 70 cm) that had padded doors, plastic lining (to collect scats), and were covered with a hessian sack. We checked traps before first light to minimise stress and allow animals to find shelter before daylight. Health checks included recording body mass, body condition, head and pes length, pouch occupancy, crown rump length of pouch young (CRL), and collection of fur and scat samples. We conducted health checks without sedation but with procedures to minimise handling time (generally <10 mins) and released animals at the point of capture. When non-target founders were captured, they were either given a health check or were weighed and released, depending on the timing of their next scheduled health check. In total, we recorded 29 founder captures in Trial 1, 50 in Trial 2 and 71 in Trial 3 during the establishment periods.

### Trial 1 tactics

In late February through to early March 2016, fourteen eastern quolls (female *n* = 6, male *n* = 8) were translocated to MFWS (Table 1). None of the females were carrying pouch young because mating was yet to occur in late austral Autumn to early Winter [43]. Releases were carried out immediately (i.e., animals were transported to ACT, underwent health assessments, and were released on the same day) from a cotton bag in randomised locations within MFWS. Releases occurred at night to minimise stress and to provide maximum time to explore MFWS and find a den before first light. No supplementary food was provided.

**Table 1:** Tactics employed for three trial reintroductions of the eastern quoll (*Dasyurus viverrinus*) to Mulligans Flat Woodland Sanctuary, ACT Australia from 2016–18. Tactics were organised as per the Translocation Tactics Classification System (Fig 1, Batson *et al*. 2015).

Tactic focus	Tactic group	Tactic type	Trial 1 (2016)	Trial 2 (2017)	Trial 3 (2018)	Rationale
Animal	Selection	Genetic	Captive and wild founders	Captive and wild founders	Wild founders	Captive and wild founders were released in Trials 1 and 2 to test the effect of origin on reintroduction success. No significant effect was found, so only genetically unique wild founders were released in Trial 3.
		Demographic	Males and females	Females only, preferably carrying young	Females only, preferably carrying young	Males have larger home ranges than females, which may have resulted in their elevated escapes in Trial 1. Females invest in natal dens, limiting their dispersal [43]. Females carrying young were preferred for Trials 2 and 3.
	Pre-conditioning	Behavioural	No behavioural assays	Behavioural assays	No behavioural assays	Behavioural assays were conducted before releases in Trial 2 (*in analysis*).
		Physiological	Pre-mating releases	Post-mating releases	Post-mating releases	Elevated hormones, and associated stress, can be experienced in breeding eastern quolls [43].
	Release design	Population size	14 founders	13 founders	8 founders	Number of founders released was dependant on availability.
	Post-release	Intervention	Limited captures following birth of young	Regular captures	Regular captures	We limited captures of females with pouch young in Trial 1 out of caution. In Trial 2, weight losses necessitated regular captures to ensure weight was regained. In Trial 3 GPS collar issues necessitated regular captures.
Environment	Pre-conditioning	Threat control	Fox control limited	Fox control intensified	Fox control intensified	Fox control was intensified outside the fence to give escapees the best chance of survival until retrieval.
			Hot-wire installed	Hot-wire modified	Hot-wire modified	The voltage of the internal hot-wire was modified following injuries to animals which contacted the wire.
			Baffles installed	Baffles present	Baffles present	Baffles (metal sheets) were installed at ‘weak points’ inside the fence to discourage escapes.
	Release design	Spatial configuration	Randomised release sites	Centralised release sites	Centralised release sites	Release sites were changed to central locations so that founders would be likely to encounter food resources, preferred den sites, other eastern quolls, or other features of interest, before the exclusion fence.
		Delayed/imm-ediate release	Immediate release (bag)	Delayed release (box)	Delayed release (box)	Releases in Trials 2 and 3 were conducted from a box to manage stress [48].
	Post-release management	Resource augmentation	No supplement feeding	Supplement feeding	No supplement feeding	Low weights were observed in Trial 2. Supplementary food was deposited into dens until weights stabilised.

Mt Rothwell refers to Mt Rothwell Biodiversity Interpretation Centre, VIC Australia.

### Data analysis

To answer our questions, we fitted a series of generalized linear models (GLMs) on five datasets comprising of one record per animal (Table 2). Response variables included effective survival (*probability of survival*) and post-release dispersal (*proportion of days moved* between dens and mean *distance moved between dens* (m)) and formed our criteria for reintroduction success. Eastern quolls that escaped the sanctuary or were transferred to another facility were treated as deceased in analyses, so we report here on ‘effective’ survival (henceforth “survival”), which does not include the survival of those escapees that were retrieved from beyond the fence. GLMs were fitted using R version 3.4.0 [49,50], model fit was assessed using chi-square tests of significance, and post-hoc Tukey’s tests were used to identify significance differences between groups using the *lsmeans* package in R [51]. We logit-transformed the data to satisfy the assumption of normality.

**Table 2:** Models, datasets used and effect sizes for probability of effective survival, den sharing and proportion of days moved between dens for eastern quolls (*Dasyurus viverrinus*) reintroduced across three trials to Mulligans Flat Woodland Sanctuary, ACT Australia from 2016–18. ‘df’ refers to residual degrees of freedom.

Response	Predictor	Dataset	Rationale	*n*	df	*p*	Figure
Probability of survival	Trial	1	Included all founders translocated	35	32	0.006	2A
	Origin	1	See 1 above	35	33	0.885	-
	Sex	1	See 1 above	35	33	0.001	2B
	Den sharing	2	Excluded the six founders which did not survive for more than 7 days	29	27	0.133	-
	Presence of pouch young	3	Excluded males	21	19	0.510	-
Den sharing	Trial	2	See 2 above	29	26	0.304	-
	Origin	2	See 2 above	29	27	0.821	-
	Sex	2	See 2 above	29	27	0.363	-
Probability of survival	Proportion of days moved	2	See 2 above	29	27	<0.001	2C
	Mean distance moved between dens (m)	2	See 2 above	29	27	0.182	-
Proportion of days moved	Trial	2	See 2 above	29	26	<0.001	3A
	Origin	2	See 2 above	29	27	0.146	-
	Sex	2	See 2 above	29	27	0.006	3B
	Den sharing	2	See 2 above	29	27	0.049	3C
	Presence of pouch young	4	Excluded males and one female which did not survive for more than 7 days	20	18	0.366	-

We divided the data into four datasets to reflect the number of founders that were appropriate for each analysis (Table 2). For example, for analyses involving *probability of survival*, *trial*, *origin* and *sex*, we included all founders translocated (dataset 1), whereas for analyses involving *presence of pouch young*, males were excluded from analyses (datasets 3 and 4, Table 2). Model selection analyses were not appropriate, either because most models were fitted with differing underlying datasets, or because those predictor variables that did use the same datasets (e.g. trial and sex) were confounded (e.g. male founders in the *sex* predictor were nested in Trial 1).

#### 1. Did adapted tactics improve reintroduction outcomes?

To determine whether survival differed between trials, we fitted a binomial GLM with a logit link function using *probability of survival* (survived = 1, deceased = 0) as the response variable and *trial* as the predictor variable (dataset 1, Table 2). To determine the factors which influenced survival, we fitted a series of binomial GLMs using *probability of survival* as the response variable and *origin* (dataset 1), *sex* (dataset 1), *den sharing* (whether a founder was found den sharing with another. founder during the establishment period, dataset 2) and *presence of pouch young* (dataset 3) for females as the predictor variables (Table 2). Den sharing in eastern quolls may be a function of sex and sociality [43] and could therefore encourage site fidelity, so we chose to include this behaviour as a predictor variable for post-release survival and dispersal, as well as a response variable for *trial*, *origin* and *sex* (dataset 2).

#### 2. What mechanisms drove improvements to reintroduction outcomes?

To determine whether post-release dispersal influenced survival, we fitted a binomial GLM using *probability of survival* as the response variable and *proportion of days moved* between dens and mean *distance moved between dens* (m) as the predictor variables (dataset 2, Table 2). For these analyses, only animals which remained alive for 7 days or more (*n* = 29) were included, and records that did not have a consecutive location from the previous day were discarded from analyses to ensure continuity of data between days post-release. To determine the factors that influenced post-release dispersal, we fitted a series of binomial GLMs with a logit link function using *proportion of days moved* as the response variable and *trial* (dataset 2), *origin* (dataset 2), *sex* (dataset 2), *den sharing* (dataset 2), and *presence of pouch young* (dataset 4) as the predictor variables (Table 2).

## Results

### Trial 1

To reduce the likelihood of escapes, an 11.5 km electric wire and baffles (metal sheets) were installed on the internal side of the sanctuary fence (Fig 1 and Table 1). However, four eastern quolls escaped from MFWS within the first two days of Trial 1. Daily radiotracking enabled escaped founders to be located and returned inside the fence if found in good condition. Serial escapees and founders in poor condition were transferred to Mt Rothwell.

Four (28.5%, female *n* = 3, male *n* = 1) eastern quolls survived the Trial 1 establishment period. Of the remaining ten founders, two were found dead within MFWS, one was transferred to Mt Rothwell due to poor condition and seven escaped (*n* = 3 female, 4 male). Of those that escaped, two were found dead, three died under observation from injuries sustained during and after escape, and two were retrieved alive and released back into MFWS. One male was transferred to Mt Rothwell due to poor condition. Following the establishment period, the three surviving females bore an estimated 18 young.

### Trials 2 and 3

#### Genetic selection

No significant differences in probability of survival (*p* = 0.546) or proportion of days moved between dens (*p* = 0.577, Table 2) were observed between captive and wild founders in Trials 1 and 2. As wild-caught eastern quolls from Tasmania have the potential to contribute unique genetic material which may not be represented within captive populations, we prioritised maximising genetic diversity and translocated only wild-caught female founders in Trial 3 (*n* = 8), all of which had pouch young.

#### Demographic selection

Increased aggression may be responsible for dispersal and mortality of males during the mating season [43]. Mortality of male eastern quolls was greater than females in Trial 1. In response, we adopted the tactic of translocating only adult females in Trial 2, preferring those that were carrying pouch young (*n* = 7 out of 13). This tactic allowed us to introduce new male and female juveniles (as pouch young) sired by either captive or wild Tasmanian males, and avoid the elevated male mortality and dispersal observed in Trial 1. Interestingly, this tactic may contribute to greater genetic diversity in founders because members within each litter may be sired by different males, as demonstrated in the closely-related northern quoll (*Dasyurus hallucatus*, [52]) and spotted-tailed quoll (*Dasyurus maculatus*, [53]).

#### Behavioural pre-conditioning

In Trial 2, we delayed the release of founders so that we could undertake behavioural assays (*in analysis*). Captive founders were translocated 13–22 days prior to the translocation of wild founders (Fig 1 and Table 1). During assays, founders were provided with an individual ‘den box’ with nesting material inside, which also served as their release box. This was intended to encourage habituation with the den boxes by the time of their release to reduce stress. Behavioural assays were not conducted in Trial 3, but we did continue to use the den box tactic.

#### Physiological selection

Greater numbers of male escapes may have been exacerbated by the timing of release because eastern quolls experience elevated reproductive hormones (e.g., luteinising hormone and testosterone) between March and June [43]. This stimulates greater mobility and increased aggression in males, which aids in acquiring den sites and food [43]. We suspect that females in Trial 1 may have also struggled to settle because they were being pursued by males and were also likely to have elevated reproductive hormones. To avoid these issues, releases in Trials 2 and 3 were conducted in austral Winter after the mating period, which also allowed us to translocate females with fused pouch young. This had the added benefit of reducing stress and collar fit issues (due to changes in neck size) associated with elevated hormones during the mating period.

#### Spatial configuration and delayed release

Founders in Trials 1 and 3 were released within 48 hours of acquisition, while in Trial 2 release was delayed by 11–28 days so that behavioural assays could be undertaken (*in analysis*). Following the assays, we conducted a pre-release health check for founders scheduled for release the following day and released one to three founders every two days.

While releases in Trial 1 were conducted in randomised locations (where some release sites were closer to the predator-proof fence than others), releases in Trials 2 and 3 were conducted from one of four central locations (each separated by 50 m). This tactic aimed to maximise the distance over which a founder needed to travel before encountering the predator-proof fence, while also allowing them to encounter food resources, preferred den sites, conspecifics, or other features of interest, before the fence. Founders were placed *in situ* in their den box with the door closed for one to two hours (delayed release, [54]). After last light, the door was opened from behind the den box (so the founder did not see the human) and the founder could leave of its own accord. We employed these tactics to minimise stress and to provide maximum time for founders to explore MFWS and find a den before daylight.

#### Resource augmentation

By 14 days post-release in Trial 2, four captive founders had lost 13–23% of their initial release weight. As an adaptive management intervention, supplementary food was deposited into dens in declining amounts as weights stabilised. All founders were provided with supplementary food because it could not be determined whether the intended animal ate its share due to consistent den sharing. This weight loss was not observed in Trial 3, so no supplementary feeding was provided.

#### 1. Did adapted tactics improve reintroduction outcomes?

In the female-only cohort of Trial 2, ten eastern quolls (76.92%) survived the establishment period and bore a total of 47 young. Of the remaining three founders, one was retrieved alive following escape, one was preyed upon by a fox following escape, and one was transferred to Mt Rothwell following two escapes. It is worth noting that of the fourteen escapes that occurred in the Trials 1 and 2, four were successfully retrieved at least once and re-released into MFWS and could therefore contribute to the effective population.

**Fig 2:**
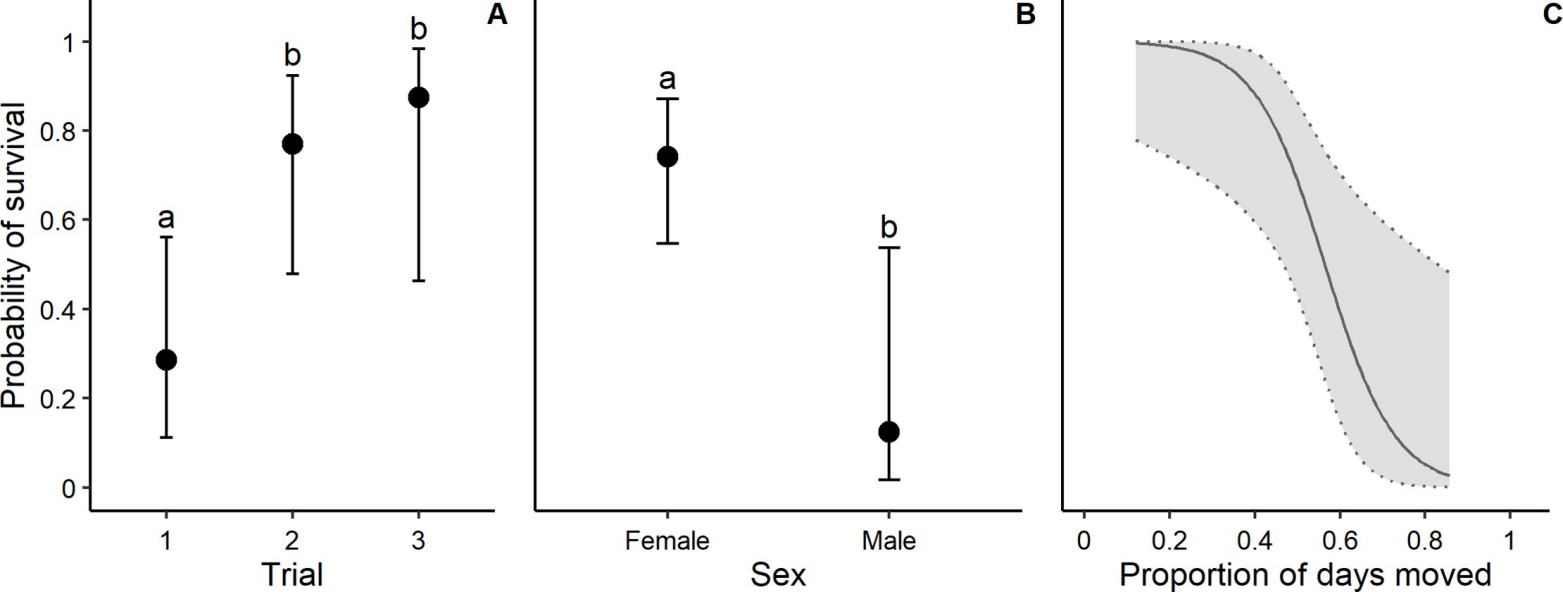
Probability of effective survival for eastern quoll (*Dasyurus viverrinus*) founders translocated to Mulligans Flat Woodland Sanctuary, ACT Australia. Survival presented by trial (2A, Trial 1 *n* = 14, Trial 2 *n* = 13, Trial 3 *n* = 8), sex (2B, female *n* = 27, male *n* = 8) and proportion of days moved between dens (2C, *n* = 29). Male animals were translocated in Trial 1 only. Error bars and dotted lines represent 95% confidence intervals for predicted values, and letters indicate significant differences (where *p* < 0.05).

In the female-only cohort of Trial 3, seven eastern quolls (87.5%) survived the establishment period and bore a total of 38 young. One founder escaped and was found to have been predated by a fox. Founders translocated in Trials 2 and 3 were significantly more likely to survive than those in Trial 1 (*p* = 0.006, Fig 2A, Table 2). Females had a significantly greater probability of survival than males (*p* = 0.001, Fig 2B).

#### 2. What mechanisms drove improvements to reintroduction outcomes?

**Fig 3:**
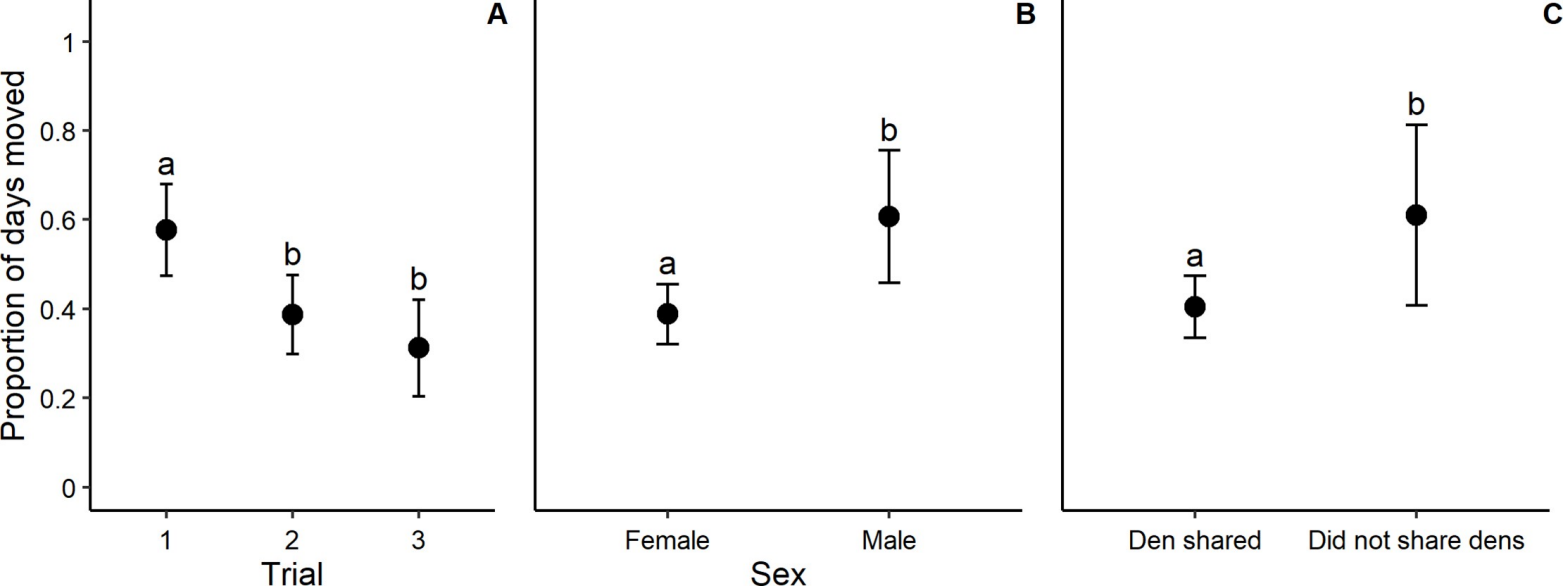
Proportion of days moved between dens for eastern quoll (*Dasyurus viverrinus*) founders translocated to Mulligans Flat Woodland Sanctuary, ACT Australia. Proportion of days moved presented by trial (3A, Trial 1 *n* = 9, Trial 2 *n* = 12, Trial 3 *n* = 8), sex (3B, female *n* = 24, male *n* = 5) and whether a founder den shared with another founder (3C). Error bars represent 95% confidence intervals for predicted values, and letters indicate significant differences (where *p* < 0.05).

Founders that moved between dens less frequently were more likely to survive the establishment period (*p* < 0.001, Fig 2C), suggesting that site fidelity impacts the probability of survival. The proportion of days where founders moved between dens was significantly lower in Trials 2 and 3 than in Trial 1 (*p* < 0.001, Fig 3A). Female eastern quolls moved between dens less frequently than males (*p* = 0.006, Fig 3B, Table 2). The proportion of days where founders moved between dens was significantly lower when an animal was found den sharing with another founder the previous day (*p* = 0.049, Fig 3C).

## Discussion

We significantly increased effective survival and limited post-release dispersal in reintroduced eastern quolls at MFWS. This was done by using a series of reintroduction trials within an adaptive management framework (outlined in Table 1 which was guided by the TTCS, Fig 1 [10]). This adaptive approach is particularly important for threatened species reintroductions, where rapid decisions are often required despite the absence of complete knowledge [7], and has been adopted worldwide for reintroductions of threatened birds [55], fish [56], mammals [57] and reptiles [58], as well as ecosystem restorations [59]. To maintain our strategies, we needed to employ tactic changes concurrently in Trials 2 and 3—naturally making a direct comparison between translocations difficult and often confounded. In addition, our inherently small (parsimonious) sample sizes did not allow us to test for potential interactions between predictor variables, reducing inferences we can make as to their combined influence on post-release survival and dispersal. Nevertheless, our results allow us to make some critical inferences about which tactics had the strongest influence on this success.

Females were significantly more likely to survive and less likely to shift to new dens between consecutive days than were males (Fig 3B). Only females are known to construct dens and use grass and sticks as nesting material [43]. It is likely that females scout for a suitable natal den, and once selected, put considerable effort into preparing and maintaining it. This would be an energetically costly behaviour, and a female would be unlikely to abandon a den she invested in. This supports our tactic of selecting females as founders in the later trials because it limited their post-release dispersal and, therefore, maximised survival in the establishment phase. This has been similarly observed in reintroductions of maternal black bears (*Ursus americanus* [60]) and elk (*Cervus elaphus* [61]), where a combination of parturition and rearing of young increased site fidelity in female founders.

Hyperdispersal, where animals that disperse great distances from the release site are unlikely to contribute to the population [62,63], can compromise the establishment [3,4,64] and monitoring [65] of reintroduced populations. Founders that survived the establishment period moved between dens significantly less over consecutive days. Interestingly, our results also indicated that den sharing reduced post-release dispersal by encouraging site fidelity, which could be a function of relatedness, sociality, and den suitability [43]. Male eastern quolls are known to avoid sharing dens outside the breeding season [38,43]. Male den sharing in Trial 1, therefore, was likely driven by mating pairs (e.g., microchip codes 8FC0 and 8DB3, see S1 Table), while den sharing between females in Trials 2 and 3 was probably driven by the need to conserve energy during Winter, as observed in smaller dasyurids (fat-tailed dunnart *Sminthopsis crassicaudata* [66], and common planigale *Planigale maculatus* [67]). We do note that den sharing with and between uncollared eastern quolls could not be detected or accounted for, so this behaviour may have been more common than observed.

It is also important to consider that founders in Trial 1 encountered an environment without conspecifics, presenting a different olfactory and social landscape compared to Trials 2 and 3. Presence of conspecifics can act as a cue for habitat quality (as per the conspecific cueing hypothesis [68]), providing indications of foraging conditions or predation risk [21,69,70]. As such, any mechanism involving conspecific attraction which may have contributed to our observed site fidelity could not be achieved at the initial release [71]. However, manipulation of visual and olfactory conspecific cueing has been used to increase settlement in reintroduced griffon vultures (*Gyps fulvus*, [30]) and black rhinoceros (*Diceros binornis*, [72]) and should therefore be considered as a tactic when planning initial releases at new sites.

Stress is an unavoidable consequence of reintroductions, and managing its effects is crucial to maximising establishment [12,73]. Increased escapes during the pre-mating period in Trial 1 led us to suspect that stress associated with immediate release may have influenced effective survival and post-release dispersal. Delayed release (where founders are housed *in situ* at the release site temporarily prior to release, [54]) can be a useful tactic for managing stress [48]. Therefore, we delayed releases in Trials 2 and 3 by two hours and made efforts to prevent the founder from seeing the researcher when their release box was opened. Remote cameras showed that some founders left the box immediately, while others explored the area around the box and returned to encounter other conspecifics after their release. This suggests that by delaying release and allowing founders to exit the box of their own accord, we were able to create a low-stress environment.

Supplementary feeding was necessary to offset observed weight losses in Trial 2, possibly due to stress associated with the pre-release behavioural assays or environmental conditions prevalent in that year. In the related yellow-footed antechinus (*Antechinus flavipes*, [74]) and southern brown bandicoot (*Isoodon obesulus*, [75]), individuals who underwent physiological experiments were found to have comparable survival and reproduction to wild conspecifics. As such, we do not consider behavioural assays contributed greatly to effective survival. While it is worth noting that supplementary feeding can alter behaviour such as migration and dispersal [28,76], founders in Trial 3 did not require this resource augmentation, and had similar levels of post-release dispersal to founders in Trial 2 (Fig 3A). Future reintroductions for this species should be prepared, therefore, to provide supplementary feed if founder weights drop significantly in response to post-release conditions; though the need for this support should not be assumed [77]. Again, this emphasises the importance of an adaptive management approach to reintroductions.

Effective survival and post-release dispersal did not differ between captive and wild founders in the Trials 1 and 2, which is consistent with results found in the reintroduction of the eastern bettong (*Bettongia gaimardi*, [11]) and releases of the Tasmanian devil (*Sarcophilus harrisii*, [78]). It is worth noting that there may be inherent differences between captive and wild founders which could impact genetic diversity, as well as performance beyond-the-fence where additional threats are present (e.g., introduced and native predators, roads, human interaction). However, these did not manifest within a predator-proof fence over our establishment period (42 days post-release).

Our study focused on the establishment phase of a reintroduction, which occurs immediately after release and is often associated with elevated mortality [79]. This is often due to the behavioural and physiological responses elicited by exposure to a novel environment, which can increase vulnerability to starvation, predation and dispersal [80]. It is worth recognising that as a founding population transitions from the establishment phase to the regulation phase [1] they are at the mercy of long-term drivers including genetic viability and habitat suitability [8,81]. This emphasises the value of monitoring reintroduced populations over the long-term to capture variability over time [8, 9, 60, 61].

Reintroduction biologists are being encouraged to adopt experimental frameworks that allow clear testing of hypotheses [7,9,21] and control for the effect of demographics, genetics and source environment [82]. However, threatened species reintroductions are inherently limited in their ability to source large sample sizes of individuals to use in experimental designs [8], and thus trial reintroductions are the most pragmatic and informative option for assessing the efficacy of tactics [17]. In our study, we observed high mortality and post-release dispersal in male eastern quolls in Trial 1, justifying a change of tactics to female-only founders released in the post-mating period in Trials 2 and 3. Rigid experimental design would dictate that we should have translocated males in each trial for comparability. With an endangered species like the eastern quoll, however, trials need to be parsimonious in the use of founder individuals to yield the maximum learning with the least number of animals [83]. Further exposure of additional animals to sub-optimal tactics for the sake of replication and control, especially when alternative tactics have shown evidence of greater success based on a multi-trial approach, is unnecessary. Nevertheless, due to these operational constraints, results should always be tested adaptively in other contexts to ensure local applicability. Based on the effectiveness of our approach, we advocate conducting reintroductions strategically within an adaptive management framework, where learnings from early trials inform tactics employed in the next [9,17]. We have demonstrated that each trial had increasing success due to the tactical changes we made.

## Conclusions

Our study demonstrates the value of fenced sanctuaries as ‘outdoor laboratories’. We were able to identify key tactics to improve reintroduction success in the absence of the introduced predators that caused their original extirpation. This provides a strong foundation for future reintroduction trials beyond-the-fence. It is important to view these mainland islands as ‘stepping-stones back to the wild, rather than reservoirs of threatened biota’ [48]. The fate of eastern quolls that escaped over the fence is a reminder of the barrier that introduced predators (particularly foxes) represent to beyond-the-fence reintroductions. The return of the eastern quoll to mainland Australia is dependent on establishing insurance populations to protect against the threat of extinction [84], while honing the reintroduction tactics that will ultimately allow the establishment of viable, free-ranging populations. Our results represent the stepping-stones by which future beyond-the-fence releases can progress.

To combat biodiversity declines worldwide, reintroduction biology will continue to develop in its applications and conservation value [33,63,64]. In contexts where there is imperfect knowledge and uncertainty about a species and its planned recipient ecosystem (for example, where the species has been absent for a long time), reintroduction success in ‘one leap’ is unlikely. Rather than viewing reintroductions as ‘all or nothing’ operations, we advocate for multiple reintroduction trials within an adaptive management framework. In this way, as we have done here, we can use our learnings from a series of initial trials, whether they were ‘successful’ at first or not, to better understand the process, build knowledge and adapt tactics that will lead to success in later trials and, ultimately, full reintroduction.

## Supporting information

S1 Table Reintroduction history. Reintroduction history of the founder eastern quolls (*Dasyurus viverrinus*) translocated to Mulligans Flat Woodland Sanctuary, ACT Australia in 2016–18.
(PDF)
