# Supplementary material for: Adapting reintroduction tactics in successive trials increases the likelihood of establishment for an endangered carnivore in a fenced sanctuary
Source: PLoS One. 2020 Jun 29;15(6):e0234455. doi: 10.1371/journal.pone.0234455 (PMC7323978; doi:10.1371/journal.pone.0234455)
Supplement: S1 Table — Reintroduction history of the founder eastern quolls (Dasyurus viverrinus) translocated to Mulligans Flat Woodland Sanctuary, ACT Australia in 2016–18. (PDF) [file pone.0234455.s001.pdf]

S1 Table. Reintroduction history of the founder eastern quolls (*Dasyurus viverrinus*) translocated to Mulligans Flat Woodland Sanctuary, ACT Australia in 2016-18.

ID	Sex	Origin	Location	Trial	Release date	Release location	Release method	Release condition	Release weight (kg)	Fate	Escapes (*n*)	Cause of death	Pouch young (*n*)	Fixes (*n*)	Den sharing (%)
8C2B	F	Captive	MtR	1	02/03/16	Pipeline Road	Bag	Good	0.835	Deceased	2	Escaped, retrieved	6	33	6
90FC	M	Captive	MtR	1	29/02/16	Main Mulligans Track	Bag	Excellent	1.215	Deceased	1	Escaped, predated (fox)		1	100
8FC0	F	Captive	MtR	1	29/02/16	Mustering Track	Bag	Good	0.785	Survived	0		7	39	26
0257	M	Captive	MtR	1	01/03/16	Old Coach Road	Bag	Good	1.120	Deceased	1	Escaped, died under observation		29	10
78F7	F	Captive	MtR	1	29/02/16	Dam Paddock	Bag	Good	0.755	Deceased	2	Escaped, died under observation	0	1	0
91DB	M	Captive	MtR	1	02/03/16	Pipeline Road	Bag	Good	1.105	Survived	0			39	5
803C	F	Wild	TAS (Pelverata)	1	29/02/16	Mustering Track	Bag	Good	1.085	Survived	0		0	40	10
8244	M	Wild	TAS (Cygnet)	1	29/02/16	Main Mulligans Track	Bag	Excellent	1.245	Deceased	0	Misadventure within sanctuary		40	10
F0C7	F	Wild	TAS (Mt River)	1	29/02/16	Main Mulligans Track	Bag	Fair	0.880	Survived	0		5	40	0
8258	M	Wild	TAS (Uxbridge)	1	01/03/16	Old Coach Road	Bag	Good	1.220	Deceased	3	Misadventure within sanctuary		1	0
81C5	F	Wild	TAS (Geeveston)	1	01/03/16	Dam Paddock	Bag	Excellent	0.875	Deceased	3	Escaped, retrieved	0	1	0
F2F4	M	Wild	TAS (Lonna Vale)	1	01/03/16	Main Mulligans Track	Bag	Excellent	1.430	Deceased	0	Escaped, died under observation		1	0
8DB3	M	Wild	TAS (Pine Tiers)	1	02/03/16	Pipeline Road	Bag	Excellent	1.420	Deceased	0	Transferred, poor condition		40	30
3AA6	M	Wild	TAS (14 Mile Road)	1	02/03/16	Pipeline Road	Bag	Fair	0.825	Deceased	1	Escaped, predated (fox)		8	13
C682	F	Captive	MtR	2	11/07/17	Central MFWS	Den box	Good	0.786	Survived	0		6	42	7
3051	F	Captive	MtR	2	28/06/17	Central MFWS	Den box	Good	0.960	Survived	0		6	42	12
5340	F	Captive	MtR	2	24/06/17	Central MFWS	Den box	Good	0.955	Deceased	1	Escaped, retrieved	0	8	0
1BEF	F	Captive	MtR	2	26/06/17	Central MFWS	Den box	Good	0.830	Survived	0		6	42	31
8849	F	Captive	MtR	2	26/06/17	Central MFWS	Den box	Good	0.880	Survived	0		4	42	43
1801	F	Captive	MtR	2	05/07/17	Central MFWS	Den box	Good	0.750	Survived	0		0	41	80
E384	F	Wild	TAS (Lonna Vale)	2	11/07/17	Central MFWS	Den box	Fair	0.746	Survived	0		6	42	12
DDE1	F	Wild	TAS (Grove)	2	13/07/17	Central MFWS	Den box	Fair	0.890	Survived	0		6	42	50
D6C1	F	Wild	TAS (Grove)	2	13/07/17	Central MFWS	Den box	Good	0.910	Survived	0		6	41	90
F9FC	F	Wild	TAS (Russell Falls)	2	17/07/17	Central MFWS	Den box	Good	0.845	Survived	0		2	42	2
E09E	F	Wild	TAS (Russell Falls)	2	17/07/17	Central MFWS	Den box	Good	0.760	Deceased	2	Escaped, retrieved	6	1	100
D897	F	Wild	TAS (Geeveston)	2	19/07/17	Central MFWS	Den box	Good	0.885	Deceased	1	Escaped, predated (fox)	6	7	0
1715	F	Wild	TAS (Geeveston)	2	19/07/17	Central MFWS	Den box	Good	0.890	Survived	0		5	42	10
3CAB	F	Wild	TAS (Blessington)	3	27/06/18	Central MFWS	Den box	Good	0.960	Survived	0		6	35	11
A98C	F	Wild	TAS (Scamander)	3	27/06/18	Central MFWS	Den box	Excellent	1.070	Deceased	1	Escaped, predated (fox)	5	28	14
414B	F	Wild	TAS (Blessington)	3	27/06/18	Central MFWS	Den box	Good	0.900	Survived	0		4	41	29
4C40	F	Wild	TAS (Scamander)	3	27/06/18	Central MFWS	Den box	Good	0.910	Survived	0		6	29	10
3E0D	F	Wild	TAS (Fingal Forest)	3	28/06/18	Central MFWS	Den box	Excellent	1.100	Survived	0		6	41	5
D0AE	F	Wild	TAS (Gladstone)	3	05/07/18	Central MFWS	Den box	Excellent	1.180	Survived	0		5	42	88
A8AD	F	Wild	TAS (Fingal Forest)	3	05/07/18	Central MFWS	Den box	Good	0.990	Survived	0		6	33	70
A810	F	Wild	TAS (Gladstone)	3	05/07/18	Central MFWS	Den box	Good	1.070	Survived	0		5	42	12

‘MtR’ refers to Mt Rothwell Biodiversity Interpretation Centre, and ‘TAS’ refers to Tasmania.
‘Bag’ refers to immediate releases from a cotton bag, and ‘den box’ releases were delayed.
‘Fixes’ is the number of radiotracking records, and ‘den sharing’ is % fixes found den sharing.
Escapee (dispersing outside the exclusion fence) and transferred animals were considered deceased for analyses.
